# Evaluating a web- and telephone-based personalised exercise intervention for individuals living with metastatic prostate cancer (ExerciseGuide): protocol for a pilot randomised controlled trial

**DOI:** 10.1186/s40814-020-00763-2

**Published:** 2021-01-11

**Authors:** Holly E. L. Evans, Cynthia C. Forbes, Daniel A. Galvão, Corneel Vandelanotte, Robert U. Newton, Gary Wittert, Suzanne Chambers, Andrew D. Vincent, Ganessan Kichenadasse, Nicholas Brook, Danielle Girard, Camille E. Short

**Affiliations:** 1 Freemasons Foundation Centre for Men’s Health, School of Medicine, University of Adelaide, Adelaide, South Australia Australia; 2 Wolfson Palliative Care Research Centre, Institute of Clinical and Applied Health Research, University of Hull, Hull, United Kingdom; 3 Exercise Medicine Research Institute, Edith Cowan University, Joondalup, Western Australia Australia; 4 School of Health, Medical and Applied Sciences, Appleton Institute, Physical Activity Research Group, Central Queensland University, North Rockhampton, Queensland Australia; 5 School of Human Movement and Nutrition Sciences, The University of Queensland, Brisbane, Queensland Australia; 6 Faculty of Health, University of Technology, Sydney, New South Wales Australia; 7 College of Medicine and Public Health, Flinders Centre for Innovation in Cancer, Bedford Park, South Australia Australia; 8 Department of Surgery, School of Medicine, University of Adelaide, Adelaide, South Australia Australia; 9 Alliance for Research in Exercise, Nutrition and Activity, Allied Health and Human Performance, University of South Australia, Adelaide, South Australia Australia; 10 Melbourne Centre for Behaviour Change, Faculty of Medicine, Dentistry, and Health Sciences, The University of Melbourne, Parkville, Victoria Australia

**Keywords:** Exercise, Metastatic prostate cancer, Behavioural change, eHealth, Computer tailoring, Aerobic, Resistance training

## Abstract

**Introduction:**

Preliminary research has shown the effectiveness of supervised exercise-based interventions in alleviating sequela resulting from metastatic prostate cancer. Despite this, many individuals do not engage in sufficient exercise to gain the benefits. There are many barriers, which limit the uptake of face-to-face exercise in this population including lack of suitable facilities, remoteness, and access to experts, significant fatigue, urinary incontinence and motivation. Technology-enabled interventions offer a distance-based alternative. This protocol describes a pilot two-armed randomised controlled study that will investigate the feasibility and preliminary efficacy of an online exercise and behavioural change tool (ExerciseGuide) amongst individuals with metastatic prostate cancer.

**Methods:**

Sixty-six participants with histologically diagnosed metastatic prostate cancer will be randomised into either the 8-week intervention or a wait-list control. The intervention arm will have access to a tailored website, remote supervision, and tele-coaching sessions to enhance support and adherence. Algorithms will individually prescribe resistance and aerobic exercise based upon factors such as metastasis location, pain, fatigue, confidence and current exercise levels. Behavioural change strategies and education on exercise benefits, safety and lifestyle are also tailored through the website. The primary outcome will be intervention feasibility (safety, usability, acceptability, and adherence). Secondary exploratory outcomes include changes in physical activity, quality of life, sleep, and physical function. Outcomes will be measured at baseline and week 9.

**Discussion:**

The study aims to determine the potential feasibility of an online remotely monitored exercise intervention developed for individuals with metastatic prostate cancer. If feasible, this pilot intervention will inform the design and implementation of further distance-based interventions.

**Trial registration:**

ANZCTR, ACTRN12614001268639. Registered 10 December 2018, https://anzctr.org.au/ACTRN12618001979246.aspx

**Supplementary Information:**

The online version contains supplementary material available at 10.1186/s40814-020-00763-2.

## Introduction

Prostate cancer is one of the most common cancers diagnosed amongst men worldwide [1]. Of those diagnosed, approximately 10–20% will present with metastatic disease at the time of diagnosis, and an additional one in five will progress from localised to metastatic disease despite treatment [2, 3]. Once prostate cancer has metastasized, the 5-year survival rate drops from 95 to 36% [4]. Metastatic disease and the subsequent treatments cause considerable physical and psychological burden [5]. Androgen deprivation therapy, radiotherapy and chemotherapy can result in increased fat mass, fatigue and pain as well as reduced muscle mass, bone mineral density, physical function and sexual function [2, 6]. In addition, over 80% of individuals with advanced prostate cancer will develop bone metastases, which can result in significant bone pain, pathological fractures and neurological impairments [5]. Individuals in this population have also been found to exhibit higher levels of anxiety and depression than their age-matched peers, including men diagnosed with localised prostate cancer [7–9]. Given the high burden, there is a clear need to develop interventions that help combat side effects, improve physical function and reduce overall burden in individuals with metastatic prostate cancer.

Research has demonstrated the beneficial effects from physical activity and more specifically, structured exercise interventions in individuals with prostate cancer [10, 11]. Currently, it has been suggested that exercise can play an important role in symptom management, rehabilitation and long-term survival [5, 10, 11]. Whilst the physiological mechanisms behind this are yet to be elucidated, it is hypothesised that exercise improves immune function, modulates circulating factors (such as insulin and growth factors), reduces inflammation and improves treatment efficacy [5]. However, until recently, those with metastatic disease were discouraged from structured exercise for fear of exacerbation of symptoms or increased risk of skeletal-related events [12].

New evidence is now available to show that supervised multi-modal exercise can be safe, feasible and clinically relevant in individuals with metastatic prostate cancer [12–15]. Cormie et al. were the first to show that resistance training is both safe (no adverse events found) and tolerable (attendance 83% and compliance 93%) in this population using a pilot study [12]. Galvão et al. then implemented a multi-modal (resistance, aerobic and flexibility training) intervention and found significant improvements in physical function (mean difference 3.2 points; 95% confidence interval (CI) = [0.4, 6.0]; *P* = 0.028) and muscular strength (mean difference of 6.6 kg (95% CI = [0.6, 12.7]; *P* = 0.033)) after 12 weeks [14]. Currently, the Movember GAP4 INTERVAL trial is examining overall survival in individuals completing a vigorous-intensity face-to-face multi-modal exercise programme [16].

Despite the mounting evidence regarding the benefits of supervised multimodal exercise for this population, many do not engage in sufficient physical activity to obtain health benefits. Zopf et al. found that only 20% of patients achieved 50–149 min per week of self-rated moderate to vigorous aerobic activity, and 29% of patients achieved ≥ 150 min [17, 18]. This is despite evidence that 92% of individuals with advanced cancer being interested in becoming more active [19]. Barriers to exercise in this population include general exercise barriers such as the lack of suitable facilities, remoteness, motivation and access to experts, as well as disease-specific barriers such as significant fatigue, urinary incontinence, mood, high levels of other medical commitments and lack of education regarding exercise for individuals with prostate cancer [8, 20]. It is currently unknown how many Australian individuals with metastatic prostate cancer receive individualised exercise advice.

Home-based exercise programmes offer a feasible alternative to counteract some of the obstacles to on-site exercise interventions because they may be able to reduce location constraints, financial and time limitations [21, 22]. However, current research indicates that these interventions produce smaller effect sizes in cancer populations on both quality of life and physical function when compared to face-to-face exercise [10, 23]. Lack of supervision and personalised support, reduced intervention adherence and limited individualisation are all possible causes of this discrepancy [10, 24, 25].

The use of technology in distance-based health care, otherwise known as eHealth, may help to improve the capacity of distance-based programmes. For example, web-based platforms have the ability to prescribe and demonstrate tailored exercise plans, provide tailored behavioural change advice, facilitate self-monitoring and support communication with healthcare professionals. Much of this can be achieved in an automated fashion using computer-tailoring techniques, thus allowing for personalization at a low-cost [26]. Reviews of digital health interventions for behaviour change suggest that some level of human support is important for efficacy [27, 28]. E-Health interventions utilising some of these techniques to support prostate, colorectal, breast and leukaemia cancer populations have already been trialled with good effects in terms of improved physical activity levels and reduced sedentary behaviour [24, 29–32]. However, the extent to which these techniques are acceptable, safe and potentially effective for supporting individuals with metastatic prostate cancer, given their unique needs and risk profile, is unknown. Our study seeks to address this gap by conducting a pilot evaluation of ExerciseGuide, a web-based and telephone supported personalised exercise intervention designed for individuals living with metastatic prostate cancer.

The primary objective of the trial is to assess key areas of uncertainty regarding the use of ExerciseGuide (and other similar programmes) in future practice and research, including issues relating to feasibility, safety and potential for efficacy. Publication of this protocol aims to ensure transparency around pre-specified criteria for success, aid replication of study and intervention methods and inform interested parties of the upcoming trial results.

## Methods

### Study design

This study is a two-arm pilot randomised control trial with participants randomised into either the intervention group (8 weeks) or a wait-list control group. Mixed evaluation methods will be used, with main outcomes assessed using questionnaires and accelerometers at baseline and post-intervention, and via a qualitative interview at post-intervention only. A sub-group of participants will be invited to complete physical function testing at baseline and post-intervention.

**Fig. 1:**
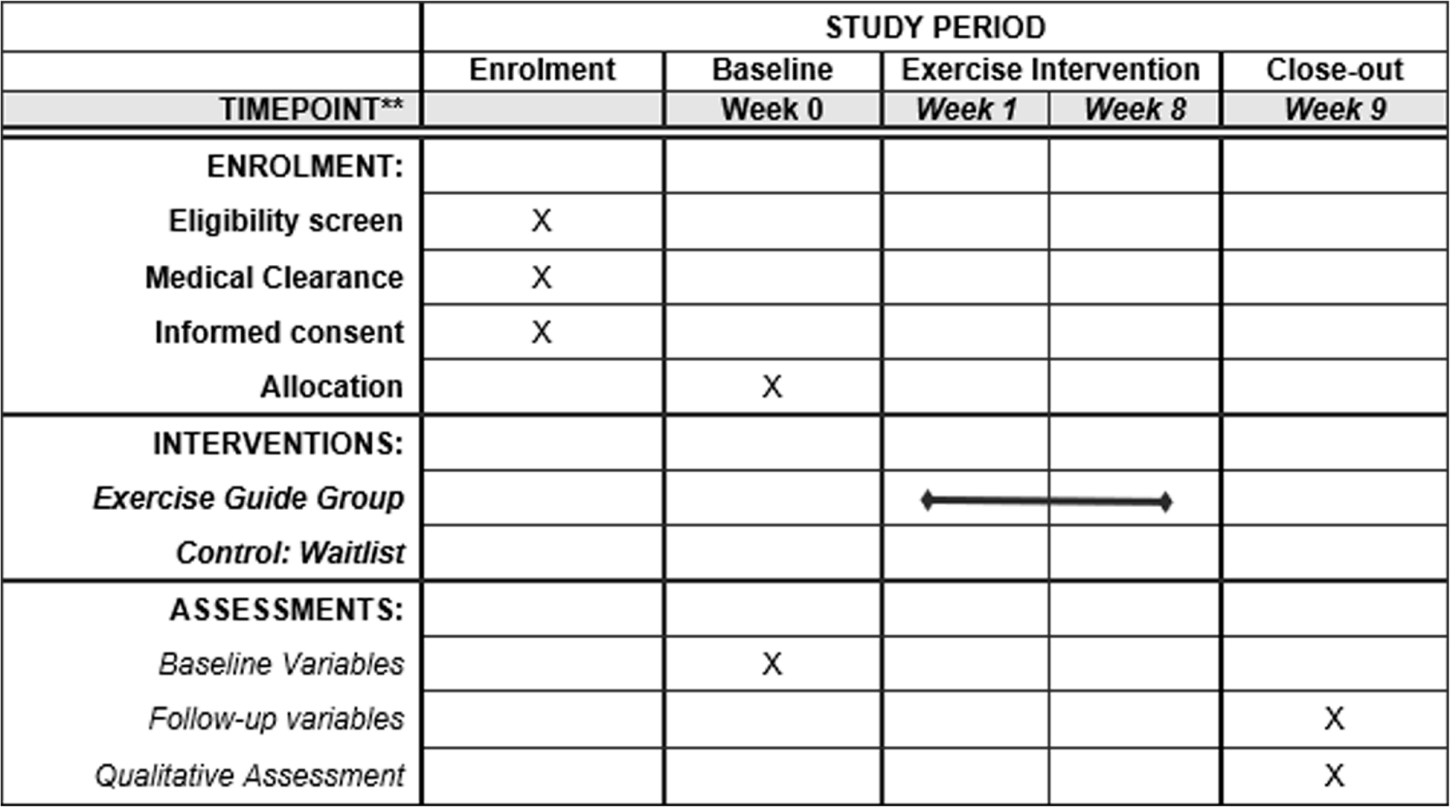
SPIRIT figure of enrolments, interventions and assessments

The trial has been prospectively registered on the Australian New Zealand Clinical Trials Registry (http://www.anzctr.org.au): ACTRN12614001268639 and ethical clearance were obtained by the University of Adelaide Research Ethics Committee (H-2018-153). To aid replication, study materials, such as the participant information sheet and data request forms from physicians, are available via the open science framework (https://osf.io/jfmy2/). The reporting of the study protocol is in accordance with the Standard Protocol Items: Recommendations for Interventional Trials (SPIRIT) guidelines [33]. The SPIRIT figure outlining the time procedure is shown Fig. 1.

### Pre-established criteria

Feasibility of conducting a larger-scale evaluation will be interpreted based on the following:

1. The recruitment goal has been reached (66 participants in 10 months).
2. Behaviour change data are collected for ≥ 75% of participants recruited within the study.
3. Physical functioning data (collected on a subset of participants) can be collected for ≥ 75% of participants that (a) reside within 30 km from a study testing site and (b) are invited to complete testing.

The success of the intervention will be interpreted based on the following:

1. The acceptability of the intervention is satisfactory (score of ≥ 20 on the client satisfaction questionnaire) [34].
2. The system usability is satisfactory (score of ≥ 68 on the software usability scale) [35].
3. There is no grade 3+/life threatening or severe adverse events attributed to participating in the intervention using the Common Terminology Criteria for Adverse Events V.5.0 grading criteria.
4. There is evidence of meaningful participation in either aerobic activity and or resistance-based activity in the intervention group relative to the wait-list control. As the exercise prescription will be personalised, it is difficult to specify an average cut-point; however, we anticipate a between-group difference of at least 30 min of aerobic activity and/or one session of resistance training. This would be indicative of behaviour change that is in line with the minimum level of exercise progression prescribed in our intervention and should also allow us to detect differences in maintenance of activity (equivalent to one session per week) amongst those that enter the study already completing some exercise.

The trial team will determine if progression to a larger scale evaluation is warranted based on the criteria and will work together to revise aspects of the protocol if problem areas are identified (e.g. if recruitment is slow the study may still progress if additional recruitment sites can be secured). The process evaluation will be used to inform improvements to the intervention if usability, acceptability or potential for efficacy issues are identified.

### Study setting

This study is being conducted in Australia. Due to the distance-based nature, participants can live anywhere in the country providing they meet the eligibility criteria. Recruitment began in February 2020 and will continue for a minimum of 10 months, unless the desired sample size is reached beforehand.

### Participants and screening

**Fig. 2:**
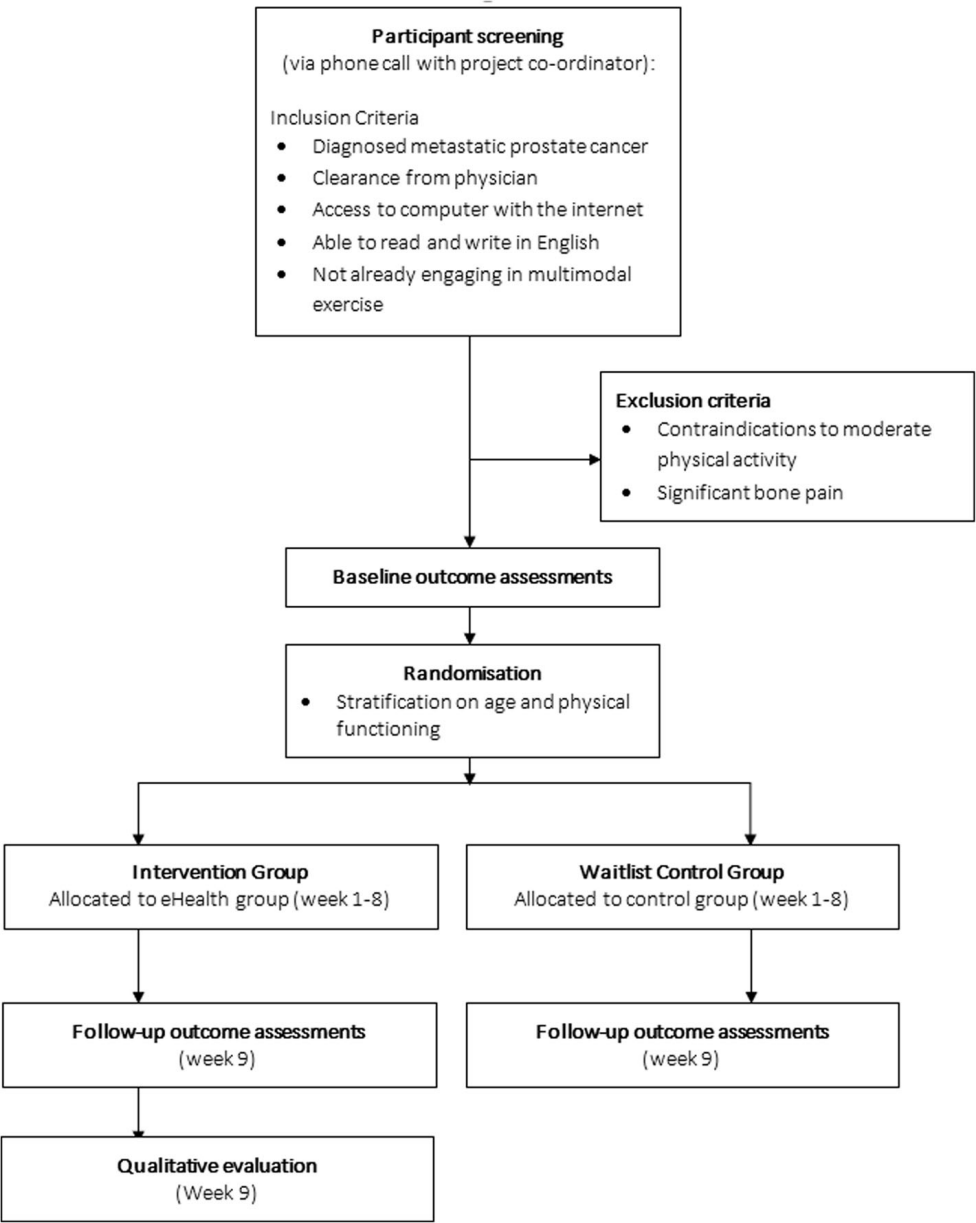
Study flow diagram

Participants are being recruited via a variety of methods. This includes dissemination of study information to potential participants within Australia via intermediaries (e.g. urologists, oncologists and nurses from the Southern Adelaide Local Health Network and Central Adelaide Local Health Network). Recruitment will also occur through the South Australian Prostate Cancer Clinical Outcomes Collaborative registry, Freemasons Foundation Centre for Men’s Health recruitment registry, Prostate Cancer Foundation Australia (e.g. Pathfinders recruitment registry and support groups), social media advertisements (e.g. on twitter and Facebook), and direct contact (e.g. presentations to consumer groups). Interested individuals are referred to the project coordinator (HE) to confirm eligibility, obtain informed consent and clearances and arrange outcome assessments. Figure 2 outlines the participant flow diagram.

To be eligible, participants must have been diagnosed with metastatic prostate cancer (as confirmed by their physician) and have medical clearance from their physician (General Practitioner, Medical Oncologist, Radiation Oncologist or Urologist). Physicians are required to provide details regarding the extent and location of metastases. Participants also need to have access to a computer with the internet, be able to read and write in English and provide written consent prior to baseline testing. Finally, to be eligible participants should not already engage in two sessions of resistance-training and 60 minutes of structured moderate-vigorous aerobic exercise per week. Participants meeting one target but not the other will be eligible.

Patients will be deemed ineligible if they have any absolute contraindications to performing moderate physical activity (resistance, aerobic and flexibility) for at least 20 min (in bouts of 5 min), up to 2 days of the week. This includes no recent serious cardiovascular events (within 12 months), unstable bone metastases, spinal compressions or acute illness and infection [36]. Participants will also be excluded from the study if they have moderate to severe bone pain (Common Terminology Criteria for Adverse Events V.5.0 grading criteria).

Participant retention strategies will include flexible scheduling for telehealth consultations, reminder calls/texts and consistent study staff. Criteria for discontinuing intervention include participant request and worsening conditions which are absolute contraindications to exercise (including unstable bone metastasis, spinal compression and significant cardiovascular events). Concomitant care will be permitted during the study and any changes in treatment will be recorded.

### Randomisation

Once baseline data have been collected, participants will be randomised into the intervention group or the control group at a ratio of 1:1. The study statistician (AV) will produce the random computer-generated number sequence in random block sizes of length 2 and 4, and will be blinded to identifying information. Stratification will occur based on age (≤ 65 years, > 65 years of age) and differences in physical function as determined by the EORTC QLC-30 (≤ 80, > 80). This is to control for potential confounders relating to age (including confidence using technology) and physical capacity. Participants will not be blinded to the primary goal of the project (evaluation of web-based tool in this population) but will not be informed of the pre-specified criteria for success.

### Exercise guide intervention

#### Intervention development

Development of the intervention (ExerciseGuide) was guided by the Intervention Mapping protocol [37], which involves a needs assessment, identification of determinants of the desired intervention outcomes and the selection of theory-based and evidence-informed strategies to target key determinants or change objectives. The process was undertaken predominantly by HE and CES, in collaboration with consumer representatives, hence drawing on expertise in exercise prescription, behaviour change and the lived experience of prostate cancer. An overview of the development process, including original research conducted and theories considered is described below.

In brief, a qualitative interview study (*N* = 18) was completed to better understand the needs and preferences of individuals in this population. This was conducted alongside a systematic review of online interventions for prostate cancer patients to determine feasibility, acceptability and efficacy, as well as factors associated with success (or failure) [38]. These studies highlighted that online supportive care interventions are acceptable in individuals with prostate cancer. Participants within the qualitative study stressed the importance of individualised exercise prescription, the need for evidence-based educational content, support and feedback to aid adherence. Importantly, participant’s accuracy of reporting metastasis location and extent was mixed, indicating further reporting measures would be required to ensure appropriate prescription.

The selection of theory to guide intervention development was informed by our original research, and evidence in the fields of exercise and health psychology more broadly [39–44]. A summary of the theories used and associated implications is provided in Supplementary Table 1. Of note, a variety of theories were considered necessary to draw upon based on the premise that exercise behaviour is guided by dual processes, and that for longer-term behaviour change to occur; it is necessary not only to address social-cognitive determinants like self-efficacy but also to address how people feel and the extent to which their behaviour change process is habit-forming in nature [41, 43]. Theory was also used to inform the architecture of the intervention and the provision of computer-tailored feedback (self-determination theory and elaboration likelihood model) [45, 46].

Once important determinants and potentially acceptable and efficacious strategies were identified based on the above research, a prototype of the intervention was developed in collaboration with consumer advisors and volunteers. This involved filming exercise videos and drafting website content and tailoring algorithms. Finally, the prototype was tested and iteratively refined in a lab-based usability and safety test (*N* = 11 patients with metastatic prostate cancer), until the intervention was considered ready for trial in the proposed study.

#### Intervention description

The intervention will use a tailored website, exercise diary and tele-coaching sessions over a period of 8 weeks to provide exercise and lifestyle support for men living with metastatic prostate cancer. Participants can drop out of the intervention at any time and concomitant care is permitted at all times.

##### Tailored website

**Fig. 3:**
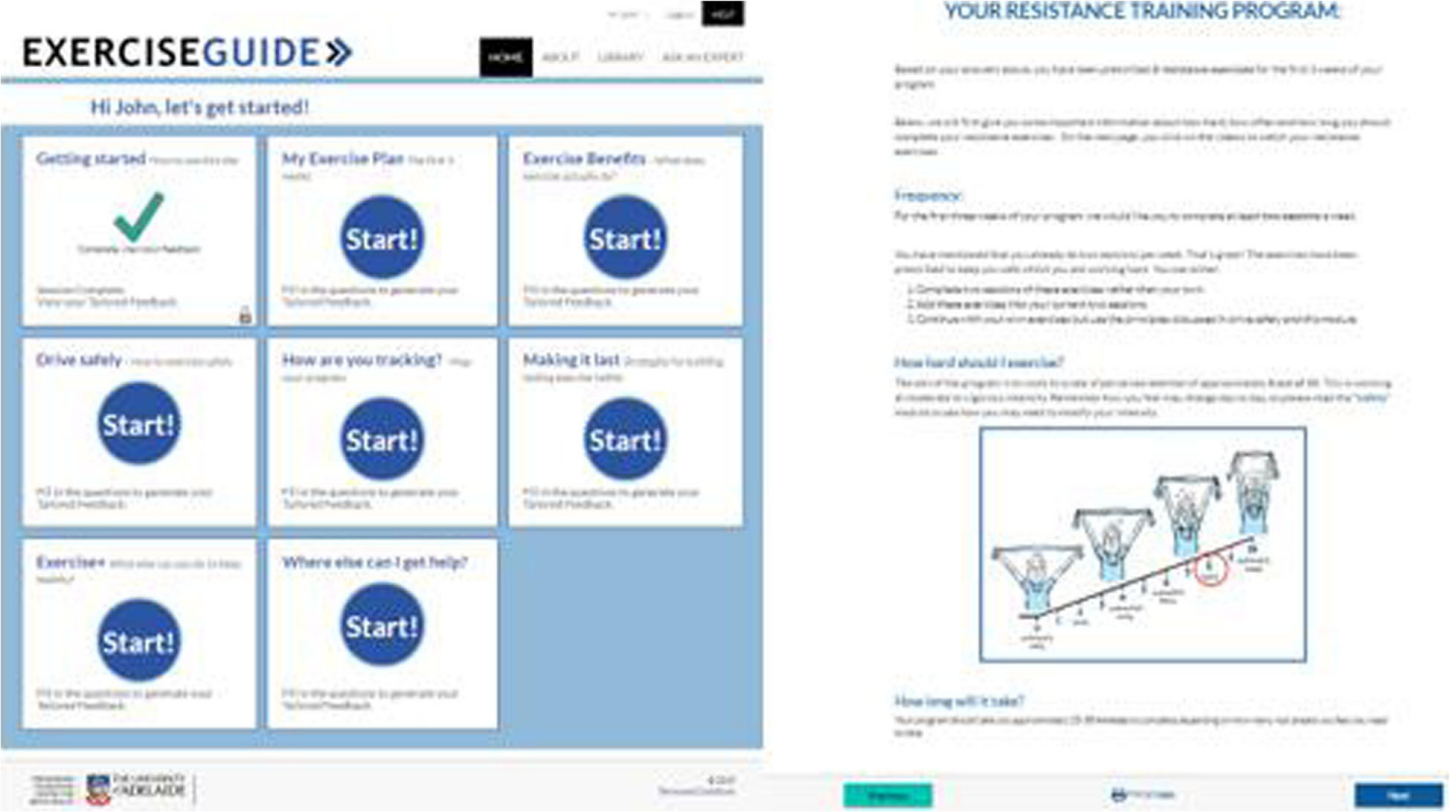
Screenshot of the Exercise Guide home page and one page of the My Exercise Plan module

The intervention group will be given access to the tailored website (www.exerciseguide.org.au) for a period of 8 weeks (Fig. 3). The tailored content, delivered via modules, will be adjusted based on individual characteristics through an automated computer process. This approach (known as computer-tailoring) leads to the delivery of more personally relevant information, which increases message safety and efficacy [21, 47, 48].

Modules include evidence-based information on the safety and benefits of physical activity, an individually tailored exercise programme, behaviour change information, self-monitoring, lifestyle information and other resources of use (Table 1). Based on the previous qualitative work in this area, participants will receive access to all modules as soon as they complete the initial introduction module and are free to complete the modules in order of personal priority.

**Table 1:** Overview of tailored modules included in 8-week intervention (page 10)

Module title	Module goal(s)	Tailoring variable	Mechanisms of action
**Getting started** (how to use this site)	Introduce the programme, including how to navigate the site.	Nil.	• Self-efficacy
**My exercise Plan** (week 1-3)	Provide individualised exercise prescription to participants.	The aerobic training component of the study is based on metastases location, current aerobic modes, current duration and frequency completed and pain levels. Self-reported ability which includes current fatigue levels, experience and confidence, will also be taken into account (14). Resistance exercise prescription is be tailored to the individual based on metastasis location pain or injury location, current fatigue levels and both resistance training experience and confidence. Favoured modes of exercise and access to equipment is taken into account for both aerobic and resistance training.	• Self-efficacy • Intentions
**My exercise plan** (week 4-8)	Progression of exercise prescription on current exercise levels.	Current aerobic exercise levels (current duration and frequency completed). Current resistance training levels (session frequency and percentage of sets and reps completed) Repeated questions from My Exercise Plan (week 1-3) with previous answers. Participants asked to re-evaluate their answers and change if needed.	• Self-efficacy • Intentions
**Exercise benefits** (what does exercise actually do?)	Help men to develop a deeper understanding of the benefits of exercise, and how these benefits accrue. Highlight benefits that are personally relevant. Strengthen intentions to participate in structured exercise.	Health issues that may be improved through exercise (e.g. fatigue, poor sleep, muscle weakness) Exercise types currently participated in (aerobic and resistance).	• Outcome expectations and identified regulation of motivation. • Proximal intentions (goals)
**Drive safely** (how to exercise safely)	Provide men with tailored information regarding safety implications to promote educational empowerment. Provide an understanding of when exercise should be terminated to avoid risk of injury.	Cancer-specific considerations that may impact the safety of exercise prescription; specifically, current treatment/disease side effects (e.g., fatigue, neuropathy) and treatments (e.g., chemotherapy, androgen deprivation therapy). Co-morbidities (e.g., diabetes, osteoarthritis) were also taken into account [10, 49].	• Self-efficacy • Outcome expectations/knowledge
**How are you tracking?** (map your progress each week)	Facilitate self-monitoring of exercise behaviours and exercise outcomes, with the aim of strengthening self-regulation.	Date, frequency of exercise, satisfaction with goal, motivation, planning score, habit scores, perceived fitness and overall fatigue, mood and pain.	• Self-efficacy • Self-regulation • Intentions
**Making it last** (strategies for building lasting exercise habits)	Support the adoption and maintenance of health-enhancing exercise behaviours.	Structured exercise programme status (interested but not commenced, commenced but finding it hard to adhere to, commenced and finding it easy to adhere to), barrier self-efficacy, exercise planning behaviours, automaticity of exercise.	Adoption • Self-efficacy • Self-regulation • Intentions Maintenance • Intrinsic motivation • Enjoyment • Automaticity (habits)
**Exercise +** (what else can you do to keep healthy?)	Increase health literacy regarding other lifestyle factors that may impact on health and quality of life besides structured exercise. Provide links to further information. Provide men exhibiting high distress with information on where to find help.	Current diet, sitting time, alcohol consumption, sleep quality, hot flushes, distress.	• Increasing knowledge • Sign-posting • Competence
**Where else can I get help?**	Facilitate access to additional support needed in order to improve lifestyle behaviours and quality of life.	Topics of interest (diet, exercise, distress, sleep, symptom management, clinical trials), preferred forms of help (e.g. guidance from a professional, booklets), and interest in advice specific to Aboriginal or Torres Strait Islander people, people who have English as a second language and/or people in the LGBTIQ community.	• Increasing knowledge • Sign-posting • Human support

##### Web-based exercise prescription

Participants are individually prescribed an 8-week multi-modal (resistance, aerobic and flexibility) programme based upon the conservative prescription used in Galvão et al. (Table 2) and isometric spinal exercise prescription applied in Rief et al. [14, 15]. Exercise prescription variables can be autoregulated by re-completing module assessment questions using the links on the homepage. The intensity or volume of session regulated can be modified by participants modifying their fatigue ratings, pain levels and confidence. Resistance exercises which produce pain can also be changed by adjusting the movement-based questions (e.g. Do you experience pain when you bend or straighten your elbow whilst holding a heavy item?). Participants are educated on autoregulation via the safety and exercise plan modules and programme modification through computer-tailoring is discussed in the initial tele-coaching session).

**Table 2:** Modified multimodal exercise prescription for individuals with bone metastases [14]

Metastasis Location	Resistance			Aerobic		Flexibility
	Upper	Trunk	Lower	WB	NWB	
Proximal humerus		^✓^	^✓^	^✓^	^✓^	^✓d^
Cervical spine	^✓a^		^✓^	^✓^	^✓^	^✓c^
Thoracic spine/ribs	^✓a^		^✓^	^✓^	^✓^	^✓c^
Lumbar spine	^✓^		^✓^		^✓^	^✓c^
Pelvis	^✓^	^✓^	^✓b^		^✓^	^✓c^
Proximal femur	^✓^	^✓^			^✓^	^✓e^

*WB* weight bearing (walking); *NWB* non-weight bearing (water walking; cycling)

^✓^Target exercise region

^a^Exclusion of shoulder flexion/extension/abduction/adduction—inclusion of elbow flexion/extension

^b^Exclusion of hip extension/flexion—inclusion of knee extension/flexion

^c^Exclusion of spinal flexion/extension/rotation

^d^Exclusion of elbow flexion/extension

^e^Exclusion of knee flexion/extension

##### Resistance-based component

**Fig. 4:**
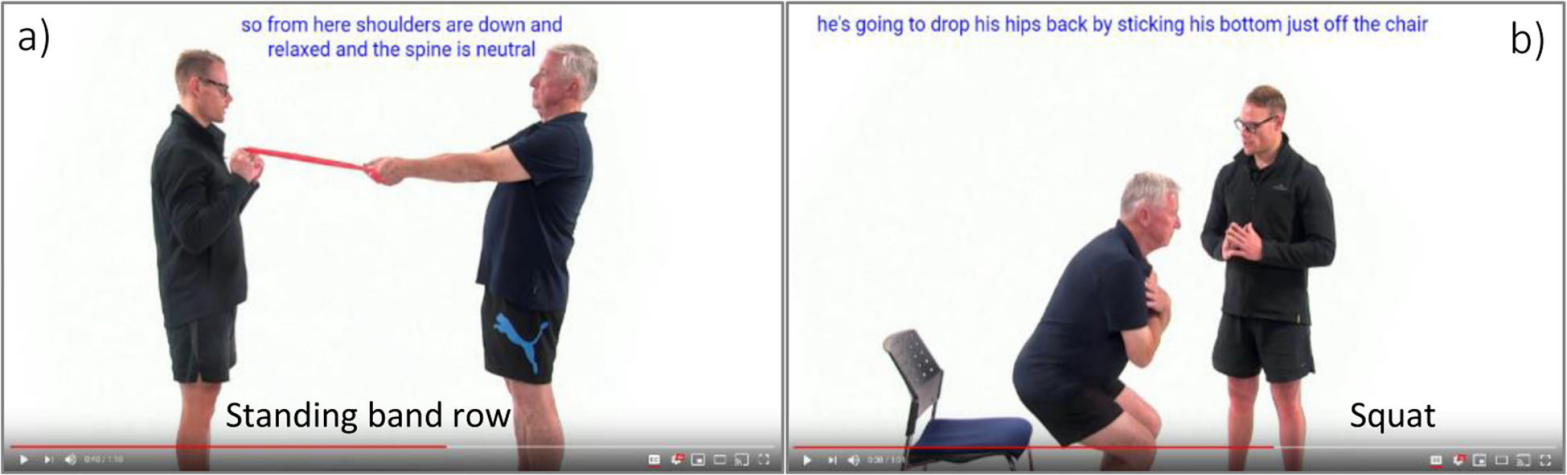
Example of the video demonstrations provided in Exercise Guide website, (**a**) standing band row and (**b**) squat

There are twenty-seven possible resistance exercises available for prescription (Supplementary Table 2), which have been used in Galvão et al. and Rief et al. [14, 15]. However, dependent on the algorithm, participants will be prescribed between three and eight exercises. Unpublished usability testing on eleven men reported a median of six exercises prescribed per person. Each resistance exercise will be accompanied by a video demonstration (see Fig. 4) and written exercise instructions to aid both safety and efficacy. Participants will be mailed four resistance exercise bands of different loads, which match the resistance bands used within the videos as well as a door anchor to help them to complete the exercises prescribed. Moreover, participants with access to home-based or gym resistance training equipment (e.g. dumbbells) will be encouraged to replicate the exercises with their equipment if possible. Eccentric and concentric phases are performed over a period of 2 seconds each to reduce peak forces transmitted to the skeleton [12–14].

**Fig. 5:**
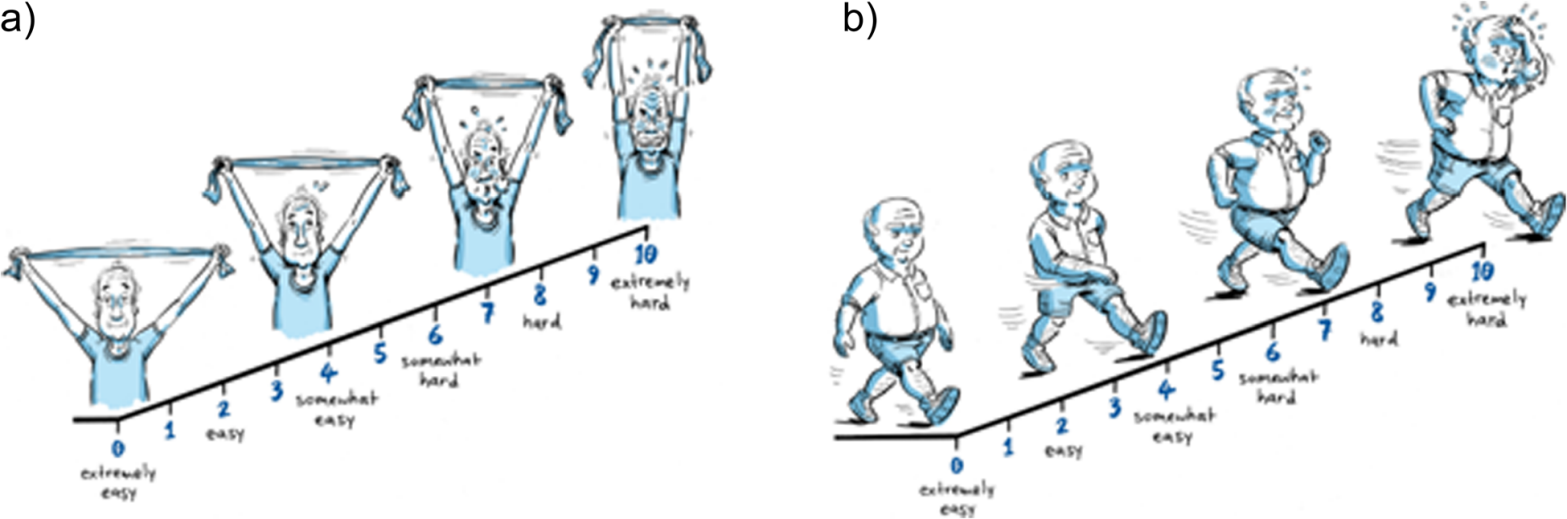
Modified illustrations of the OMNI exercise scale of perceived exertion, (**a**) resistance training, (**b**) aerobic training

Intensity will be prescribed individually using the OMNI resistance exercise scale of perceived exertion (Fig. 5) and will range between seven to eight out of ten (moderate-vigorous intensity) as determined by current fatigue levels and both resistance training experience and confidence [50]. This variability has been built in to help balance adherence with the stimulus needed to see clinically relevant outcomes. A 12- to 8-repetition maximum has been prescribed for two to three sets per exercise (Table 3). Self-reported questions at the end of week three will allow researchers to monitor compliance. Participants unable to comply to at least 80% of the prescription (based on total sessions completed and compliance to volume prescribed) will be given a modified prescription for the last five weeks (Supplementary Table 3) to ensure the participant does not increase repetitions or sets too rapidly. To encourage progression, participants will be instructed to increase the resistance of the upper and lower body exercises by self-assessed 5-10% if the rate of perceived exertion standards were exceeded in each exercise set completed [14].

**Table 3:** Resistance training prescription

Exercise type	Volume of exercise prescribed (sets x repetitions)			
	Week 1	Week 2	Week 3	Weeks 4-8
Upper body exercises	2 × 12	2 × 12	3 × 12	2-3 × 12-8 (see Additional file 3 for specific prescription)
Trunk exercises	2 × 8	2 × 8	2 × 10	2-3 × 10-12 (see Additional file 3 for specific prescription)
Lower body exercise	2 × 12	2 × 12	3 × 12	2-3 × 12-8 (see Additional file 3 for specific prescription)

Frequency of resistance training will be 2 days per week for the first 3 weeks and then increased to three sessions if the participant was able to adhere. Participants already completing one or more sessions per week prior to the intervention starting will be required to complete at least two sessions per week of the Exercise Guide programme and will be encouraged to complete more sessions of their own prescription if they wish.

##### Aerobic-based component

Individuals with bone metastases will be prescribed either stationary cycling, water walking or walking. For individuals without bone metastases, other modes such as conventional cycling, rowing or cross trainers are also prescribed. Equipment accessibility, metastasis location, pain and preference influence what mode is prescribed. For individuals currently completing two sessions or less a week, two sessions a week will be prescribed for the first 3 weeks. If individuals are completing three or more sessions, then they will be prescribed three specific aerobic sessions. Individuals are encouraged to complete extra aerobic sessions if they feel capable. In terms of duration of sessions, participants will complete 2-3 sets of between 6 and 15 min, with rest intervals ranging from 0 to 5 min or 1 session of 30 min with no prescribed rest (Table 4). Session duration and rest intervals will be based on what activity duration the participant feels is feasible, if they are currently meeting this level and an ability score based on previous experience, levels of fatigue and confidence for completing two sessions per week. Intensity will be prescribed using the OMNI aerobic exercise scale of perceived exertion [50]. Intensity will be prescribed individually and will be between six and seven out of ten (moderate intensity) as determined by current fatigue levels and both experience and confidence.

**Table 4:** Aerobic training prescription for session duration based on participant self-assessment of exercise duration feasibility, current participation and ability

Duration feasible	Meeting duration	Ability	Aerobic prescription	Total active minutes
0-4 min	No	Low/High^a^-	2 × 3-min efforts with 3-5 min rest	6 min
0-4 min	Yes	Low/High^a^	2 × 4-minute efforts with 3-5 min rest	8 min
5-9 min	No/yes^a^	Low	2 × 5 minutes with 2 min rest	10 min
5-9 min	No/yes^a^	High	3 × 5 minutes with 2 min rest	15 min
10-19 min 10-19 min	No/yes^a^ No	Low High	2 × 10-min efforts with 5-min rest	20 min
10-19 min 20-29 min	Yes No/yes^a^	High Low	2 × 15-min efforts with 5-min rest	30 min
20-29 min 30-44 min 30-44 min 45-59 min 60 min +	No/yes^a^ No Yes No/yes^a^ No/yes^a^	High High Low Low Low	1 × 30-min effort	30 min
30-44 min 45-59 min 45-59 min 60 min +	Yes No Yes No/yes^a^	High High Low High	3 × 10-min high intensity	30 min
Unknown	No/Yes^a^	High/low^a^	2-3 × 5-10 min efforts with 2-5 min rest	10-30 min

^a^Prescription did not differ based on this variable for this prescription

Flexibility exercises will be prescribed based on previous work by Galvão et al. (see Table 2), which has been shown to be safe. Static stretching will involve all major muscle groups involved in the session held for 30-60 s over a period of 2-4 sets and will be prescribed via pictorial and written instructions.

##### Tele-health coaching and monitoring of progress

To keep participants engaged in the programme and ensure sufficient support, participants will also have access to an accredited exercise physiologist (HE). The role of the exercise physiologist will be to encourage uptake of the 8-week individually prescribed programme, provide feedback and monitor progress over the 8-week intervention period. Recent reviews of online digital behaviour change interventions suggest that the inclusion of human support increases the efficacy of online interventions [51]. The exercise physiologist will make contact with participants allocated to the intervention during week one of the programme (by phone or internet call). They will discuss participant goals, provide advice about how best to use the programme to achieve their goals, and offer remote monitoring of participant progress throughout the 8 weeks. Remote monitoring will involve reviewing data entered into the website by participant’s weekly and providing encouragement, feedback and advice based on performance. Contact after week one will occur via email and text messages (up to one per week), with the exception of week three, which will involve a follow-up call (by phone or internet). Participants will have the option of submitting questions to the exercise physiologist whenever they would like to via ‘the ask the EP’ (exercise physiologist) feature of the website. Responses will be sent to participants electronically where possible.

##### Additional components

*Library*. The Exercise Guide library is populated with 25 short articles written in layman’s language about different aspects of living with prostate cancer, exercise and behaviour change. Example topics include ‘Can exercise help your sexual health?’, ‘Sitting too much: What are the real consequences’ and ‘Exercise and depression: 5 tips to move your mood’.

*Diary*. A paper-based exercise diary will be provided to participants in the intervention group to self-report specific aspects of the resistance training (exercises, sets, repetitions, session rate of perceived exertion, duration, bone pain visual analogue score and general pain visual analogue scale), aerobic training (type, duration, session rate of perceived exertion, bone pain visual analogue score and general pain visual analogue scale) and stretching exercise sessions performed. This will allow researchers to monitor and report exercise completed, subjective intensity and any changes in pain levels.

### Waitlist control group

Participants randomised into the wait-list control group will complete the baseline outcome measures as seen in Table 5 and will then be asked to continue with usual care, including maintaining their current physical activity levels for 8 weeks. In week nine, wait-list control participants will repeat the outcome measures. At the end of the intervention, the control group will be sent therabands, given access to the eHealth tool for 8 weeks, complete two tele-coaching sessions and receive weekly contact from an exercise physiologist as authors felt it was unethical to deny participants access to supported treatment.

**Table 5:** Overview of measurement tools (page 17)

Outcome	Measure	Number of items	Intervention group	Control group	Collection point (week)
Acceptability	Module ratings	10	✓	x	1-8
	Website perceived personal relevance [52]	3	✓	x	9
	Client Satisfaction Questionnaire [34]	8	✓	x	9
	Perceived Environmental Supportiveness Scale [53]	15	✓	x	9
	Qualitative data (open-ended survey questions and qualitative interview)	4	✓	x	9
Usability	Software usability scale [35]	9	✓	x	9
Usage	Website usage data [54]	3	✓	x	1-8
	Number of exercise sessions	2	✓	x	1-8
Adverse events	Common Terminology Criteria for Adverse Events V.5.0 grading criteria	1	✓	✓	9
Mechanisms of action	Self-efficacy [55]	9	✓	✓	0, 9
	Outcome expectations [55]	8	✓	✓	0, 9
	Motivation type [56]	19	✓	✓	0, 9
	Social Support [55]	2	✓	✓	0, 9
	Intention [57]	4	✓	✓	0, 9
	Behavioural capability [52]	2	✓	✓	0, 9
	Habit formation [43]	4	✓	✓	0, 9
Behaviour change	ActiGraph Accelerometer [54]	1	✓	✓	0, 9
	Modified Godin-Leisure time questionnaire [12]	4	✓	✓	0, 9
	Self-rated exercise adherence [58]	2	✓	x	9
Health outcomes	European Organisation for Research and Treatment of Cancer Quality of Life-Core 30 [59]	30	✓	✓	0, 9
	Functional Assessment of Chronic Illness Therapy-fatigue subscale [60]	13	✓	✓	0, 9
	Hospital Anxiety and Depression Scale [61]	10	✓	✓	0, 9
	The Pittsburgh Sleep Quality Index [62]	14	✓	✓	0, 9
Sub-study physical function	400 m self-paced walk [14]	1	✓	✓	0, 9
	Timed up-and-go test [12]	1	✓	✓	0, 9
	Repeated chair stand [63]	1	✓	✓	0, 9
	One-repetition maximum [14]	2	✓	✓	0, 9

### Measures

#### Feasibility

##### Trial parameters

Screening, recruitment and attrition rates will all be tracked, with reasons for ineligibility, lack of interest and drop-out recorded where possible. Recruitment source will also be assessed during study enrolment. The proportion of participants with complete data for each outcome measure will be assessed, along with the number of reminders and data collection attempts.

##### Intervention parameters

The time taken to deliver coaching sessions and respond to questions asked using the ‘ask and EP feature’ will be recorded in order to provide a proxy indicator of cost of delivery.

#### Success of the intervention

##### Acceptability

The acceptability of the intervention will be assessed using a mixed-methods approach. Participant’s perceptions of module content will be assessed in real-time using a five-star rating system (1—poor to 5—excellent). All other acceptability items will be assessed in week 9. Perceived personal relevance of website content will be assessed via survey using three items designed to evaluate the success of tailoring on a 7-point Likert scale (e.g. ‘the web-based content was written with someone like me in mind’) [52]. The extent to which the coaching was perceived as motivationally supportive will be assessed using the 15-item perceived environmental supportiveness scale [53] and overall satisfaction with the intervention as a health service will be assessed using the client satisfaction Questionnaire-8 [34]. Finally, qualitative data will be collecting using open-ended survey questions and qualitative interviews exploring the pros and cons of the website, any unmet needs, and recommendations for improvement, including any suggested changes to intervention length.

##### Usability

Usability of the intervention website will be assessed using the System Usability Scale [35]. The survey consists of a 9-item questionnaire with five response options for respondents ranging from ‘strongly agree’ to ‘strongly disagree’.

##### Usage

Website usage data, including the number of logins, time on site and number of modules completed, will be assessed using Google analytics and inbuilt website tracking software [54]. Usage of the exercise diary will also be recorded in terms of number of sessions logged.

##### Adverse events

Participants will be advised to report any injuries either through the website or by calling the study project coordinator (HE). An item on adverse events will also be included in the follow-up questionnaire based on the Common Terminology Criteria for Adverse Events V.5.0 grading criteria.

##### Behaviour change

Accelerometry will be used to objectively measure weekly minutes of light, moderate and vigorous physical activity at baseline and immediately post-intervention. Data will be collected using the ActiGraph (ActiGraph GT3X, http://www.theActiGraph.com) accelerometer, which will be worn on the right hip during waking hours for 7 days with the intention of gaining at least 5 days of usable data [54]. Participants will record times the monitor was removed and wear-time will be validated using Choi et al. [64]. Triaxial data will be collected in 1-s epochs along with step counts and inclinometry. The adapted Godin leisure-time questionnaire will be used to assess self-reported aerobic and resistance based physical activity at baseline and immediately post-intervention [12]. Finally, at the immediate post-intervention follow-up only, self-rated exercise adherence will be measured using two items with an 11-point numeric rating scale (0 = strongly disagree, 10 = strongly agree). Participants will be asked to separately rate their agreement with two statements related to their adherence to their prescribed programme (overall aerobic exercise sessions, overall resistance exercise sessions) [58].

##### Health outcomes

All health-related outcomes will be assessed at baseline and immediately post-intervention. Health-related quality of life will be assessed using the EORTC Quality of Life-Core 30 (EORTC QLQ-C30), which is a validated and reliable questionnaire for quality of life in cancer patients [65]. The 30-item core survey assesses a comprehensive range of quality of life domains including functioning (physical, role, cognitive, emotional and social), symptoms (fatigue, nausea and vomiting, sleep, pain, appetite, shortness of breath), financial hardship and global health status [59].

The 13-item Functional Assessment of Chronic Illness Therapy-fatigue subscale will be administered to measure participant’s level of fatigue. The questionnaire has been demonstrated as a valid and reliable measure [60]. The Hospital Anxiety and Depression Scale will be used to evaluate changes in anxiety and depression by using two 7-item Depression and Anxiety sub-scales [61]. The reliability and validity has been shown to be acceptable in individuals with prostate cancer [66]. Additionally, the Pittsburgh Sleep Quality Index questionnaire will measure sleep quality. Psychometric evaluation of the Pittsburgh Sleep Quality Index in cancer patients has established internal consistency, reliability and construct validity [62].

#### Intervention mechanisms

The proposed behavioural change mechanisms will be assessed at baseline and week 9. This will include measuring barrier self-efficacy for resistance and aerobic activities (9 items [55]), outcome expectations (8 items [67]), motivation type (19 items [56]). Social support (2 items [55]), intentions (4 items [57]), behavioural capability (7 items [52] and habit formation (4 items [43]). Collection of this data will provide preliminary insight into if the intervention is working as expected and will also provide useful information for directing further tailoring efforts (e.g. by allowing us to examine how variable individuals are in these variables at baseline, and thus the extent of tailoring that is needed). Collection of this information will also be useful for establishing feasibility of collecting data needed for a formal mediation analysis in the main trial.

#### Sub-study outcome measures

Subgroup selection will be based on proximity to available testing sites, with all participants (both intervention and waitlist control groups) who can easily access one of our testing sites invited to complete subgroup measures). We intend to have testing sites available in Adelaide (the University of Adelaide, University of South Australia) and Melbourne (University of Melbourne).

##### Physical function

The 400-m walk will be used to assess aerobic fitness level [14, 68]. Participants will be asked to complete the walk as fast as they complete 20 laps of 20-m track. Measures of completion time (in seconds), maximal heart rate, average heart rate and rate of perceived exertion will be recorded. The timed up-and-go test and the repeated chair stand (5 repetitions) will be measured by time to completion to provide muscular power, ambulation and functional lower limb strength [12, 13, 63].

##### Muscular strength

The one-repetition maximum method will be utilised to determine muscular strength [14]. The leg extension will be used to determine lower limb strength, and the chest press will assess upper limb strength. Patients with proximal femur bone lesions will be excluded from the leg extension one-repetition maximum test, whereas those with rib, thoracic spine lesions and or humerus lesions will be excluded from the chest press one-repetition maximum test.

### Data management and monitoring

All research data will be stored on a password-protected network drive on a password-protected computer and only members of the study team will have access to this data. Questionnaire data will be recorded using a secure online data collection instrument (RedCap) with participants being sent web links at the appropriate time points. Participant contact information and name will be held in a separate file to study data, and unique ID numbers will be allocated. Only ID numbers will appear alongside outcome data. A trial steering committee (CES, DG, GK, NB) will oversee the trial. Any adverse events will be reported to the committee and the steering committee have the power the terminate the study if necessary. As a clinical oncologist and urologist sit on the trial steering committee, it was determined that a trial data monitoring committee was not required. Trial results will be submitted for publication and communicated in a relevant medical or scientific journal.

### Data analysis

#### Sample size

As this is a pilot study, which uses pre-specified criteria for success rather than a primary outcome, a formal sample size calculation was not essential [69]. Similar 2-arm pilot studies have reported sample sizes of 36 and 30 per arm for dichotomous and continuous endpoints, respectfully [70]. Similar to these studies, the enrolment target for this trial is 66 participants, 33 in each arm. Using a sample size calculation method created for pilot studies by Vichtbauer al., it was determined that the above sample size will allow us to identify feasibility problems (drop out, safety issues etc.) with a reasonable probability of occurring (i.e. a probability of 10% or greater), with a 95% level of confidence [71]. If issues are detected the trial team will review and consider if progression to a larger trial is warranted and feasible.

Study data will be analysed using SPSS version 26 (IBM, Chicago, IL, USA). Descriptive statistics will be calculated for all study variables. ANCOVAs (or non-parametric equivalents) will be used to conduct between-group comparisons, with potential confounders included in the model (e.g. age, time since diagnosis, treatment status and baseline outcome assessments). Treatment effects will be estimated as covariate-adjusted mean differences between the two treatment groups at follow up. A senior University of Adelaide statistician employed by the Freemasons Foundation Centre for Men’s Health will oversee the analyses. Sensitivity analyses will be conducted to explore the impact of missing data and remove any individuals who were unable to be prescribed with exercise from the analysis.

#### Qualitative analysis

Any verbal feedback from participants will be recorded (with permission) and transcribed verbatim. A thematic assessment will be undertaken to analyse the data as completed by Braun and Clarke [72]. This approach is data-driven and involves becoming familiar with the data, generating initial codes, searching for themes amongst codes and refining the themes to fit the data better.

### Protocol changes

Future amendments to the protocol will be considered and approved by the Steering Committee and resubmitted for ethical approval. Approved amendments will be subsequently distributed to participants through the study website.

## Discussion

The primary objective of the trial is to assess key areas of uncertainty regarding the use of ExerciseGuide (and other similar programmes) in future practice and research, including issues relating to feasibility, safety and potential for efficacy. Publication of this protocol aims to ensure transparency around pre-specified criterion for success, aid replication of study and intervention methods, and inform interested parties of the upcoming trial results.

ExerciseGuide represents a novel approach to providing more geographically accessible individualised exercise prescription with distance-based multidisciplinary support. The addition of distance-based supervision, tailored behavioural change strategies and telehealth support, may improve adherence to distance-based interventions, which are currently seen as inferior in comparison to the gold standard ‘face-to-face’ intervention [23, 29]. Increasing the feeling of personalisation, connectedness, and support through eHealth has been one method to address the disparity between the two approaches. It is anticipated that an intervention of this type would not replace face-to-face interventions, but rather play a role alongside on-site interventions as an alternative where no onsite interventions can be made available due to staffing or financial shortages.

Despite the potential advantages, there are a number of implementation issues that may be encountered when delivering a programme like ExerciseGuide. These need to be mitigated in order for high-quality implementation and evaluation to occur in the future. For example, whilst the use of eHealth technologies is useful to increase accessibility to individuals in remote areas, it is important to note that internet connection and reliability are reduced in remote Australian areas [73]. Our provision of both written (printable) and video-based exercise prescription and education may improve access to those with poor Internet connections; however, those without a connection will remain without accessible support. Adherence to health behavioural change and exercise interventions is another limitation noted in the eHealth literature [26]. We have attempted to improve adherence by working with consumers to refine content and by undergoing iterative usability testing in order to improve the user experience. We have also included elements of human support which should further enhance engagement. Lastly, it is well known that recruiting older males into health interventions is difficult [74]. Numerous different recruitment strategies will be included such as mass mailing via prostate cancer registries, health service referrals and community outreach. Further to this, advertising materials were gender-specific and mass mailouts through the prostate cancer registry were personalised. Data from this trial will provide useful information regarding the success of these mitigation strategies.

Pilot trials studies of this nature can possibly reduce research waste and more significantly, they can lead to changes in development and design in order to maximise the intervention and trial characteristics for future trials [75]. Usability, acceptability, adverse events, safety, and evidence of aerobic activity or resistance-based activity participation will all be monitored to determine if progression to a full randomised control trial is worthwhile.

## Supplementary Information

**Supplementary file 1.** Theoretical tenants and strategies incorporated into the ExerciseGuide program. Tabular explanation of theories underpinning the ExerciseGuide program and examples of strategies using the theories.

**Supplementary file 2.** Resistance-based exercises prescribed within the ExerciseGuide program. Table detailing all possible resistance training exercises prescribed within the ExerciseGuide program.

**Supplementary file 3.** Modified prescription for the last five weeks for individuals unable to comply to at least 80% of resistance exercise prescription. Tabular explanation of modified resistance training exercise prescription for weeks 4-8 for individuals who were unable to comply to at least 80% of resistance exercise prescription.

## Data Availability

Not applicable.

## Abbreviations

SPIRIT: Standard Protocol Items: Recommendations for Interventional Trials
CTCAE: Common Terminology Criteria for Adverse Events
EORTC QLC-30: European Organisation for the Research and Treatment of Cancer Quality of Life Questionnaire-30 items
WB: Weight bearing
NWB: Non-weight bearing
EP: Exercise physiologist
