# Supplementary material for: Evaluating a web- and telephone-based personalised exercise intervention for individuals living with metastatic prostate cancer (ExerciseGuide): protocol for a pilot randomised controlled trial
Source: Pilot Feasibility Stud. 2021 Jan 11;7:21. doi: 10.1186/s40814-020-00763-2 (PMC7798256; doi:10.1186/s40814-020-00763-2)
Supplement: Supplementary file 1 — Theoretical tenants and strategies incorporated into the ExerciseGuide program. Tabular explanation of theories underpinning the ExerciseGuide program and examples of strategies using the theories. [file 40814_2020_763_MOESM1_ESM.doc]

Supplementary Table 1: Theoretical tenants and strategies incorporated into the ExerciseGuide program

| **Theoretical tenets and supporting evidence** | **Strategies** |
| --- | --- |
| *Self-determination theory* | |
| - Motivation that regulates our behaviour exists on a continuum, ranging from extrinsic (doing the activity for instrumental reasons or to obtain an outcome separate from the activity) to intrinsic (performing a behaviour because it is inherently satisfying). - Behaviours are more likely to be maintained when they are more intrinsically motivated. - Core determinants of intrinsic motivation include three basic psychological needs: autonomy, competence and relatedness. | - Autonomy: Intervention architecture promotes choice and is self-timed; Participant preferences are taken into account with exercise prescription where possible (balancing safety, efficacy); affect-regulated exercise is encouraged if participants are not enjoying their prescribed program (1). - Relatedness: The intervention provides direct access to an Exercise Physiologist twice and an option to ask questions to the Exercise Physiologist to provide a sense of attachment and personalization to the intervention. Photographs of all team members are also included on the website. Human support has been shown to enhance the efficacy of online interventions (2). - Competency: The intervention provides education, behavioural change tools and individualised exercise prescription with video demonstrations to increase skill level that is needed to help take actions that will help them achieve their goals. A positive, encouraging tone is adopted throughout and the intervention has been designed to be simple and easy to use. (3) |
| *Social Cognitive Theory* | |
| The core determinants that influence behaviour include perceived self-efficacy, outcome expectations, goals people set for themselves and strategies people have for realizing them and perceived impediments and facilitators (4). Self-efficacy and goals (proximal intentions), as well as perceived health impediments have been associated with exercise behaviour change in prostate cancer patients. (5,6,7) | - Self-efficacy: Video demonstrations and safety advice provided to improve task self-efficacy, especially for resistance-training. Exercise prescription is progressed over-time and tailored to meet individual needs. Encouragement is provided throughout to build confidence. - Outcome expectations: Comprehensive education is provided on exercise benefits focusing on both physical and mental health. Perceived costs of participating reduced by providing convenient program structure and all necessary equipment and information. - Goal and self-control strategies: A print-based exercise diary and tracking module is provided allowing participants to monitor progress. Tailored psycho-education is provided regarding implementation planning. - Impediments: Health impediments are addressed by tailoring exercise prescriptions based on functional capacity. Environmental barriers are reduced by offering a distance-based program free of charge. |
| *Habit Formation Theory* | |
| Habit formation theory states that ‘habits’ are behaviours that are triggered automatically in response to cues that have been linked with the behaviours’ performance (8). Habitual behaviour is regulated by impulsive processes, and so does not require large amounts of cognitive effort, control or even a conscious intention. Habits have been shown to predict physical activity behaviour when intentions are weak (8,9). Having an exercise habit has been associated with increased maintenance of behaviour change (8). | - Context-behaviour associations: Tailored psycho-education is provided describing current habit strength and providing information on the benefits of habits and guidance on how they are formed.   . |
| *Elaboration Likelihood Model of Persuasion* | |
| According to this information processing theory persuasion can occur via two pathways, a central route involving elaborate thinking and a peripheral route involving the use of heuristics and feelings. Persuasion can occur via either pathway but persuasion via the central route may result in more enduring attitude change. A key determinant of using the central route pathway is perceived personal relevance of the information. Need for Cognition, a personality factor also predicts tendency to use central or peripheral route processing (10,11). | - Relevance: All modules provide tailored content based on an individual assessment. This is expected to increase perceived personal relevance of the website content. - Need for cognition: Detailed explanations and source of recommendations are provided to appeal to those with moderate-high need for cognition (i.e., those who tend to enjoy effortful cognitive activities). Pictures and videos, credibility cues (e.g. university logos), and the ability to skip information and ask direct questions is designed to appeal to those with a lower need for cognition. |

**References:**

1. Parfitt G, Alrumh A, Rowlands AV. Affect-regulated exercise intensity: does training at an intensity that feels “good” improve physical health? J Sci Med Sport. 2012;15(6):548–53.
2. Santarossa S, Kane D, Senn CY, Woodruff SJ. Exploring the role of in-person components for online health behavior change interventions: Can a digital person-to-person component suffice? J. Med. Internet Res. 2018;20(4):e144.
3. Short CE, James EL, Rebar AL, Duncan MJ, Courneya KS, Plotnikoff RC, et al. Designing more engaging computer-tailored physical activity behaviour change interventions for breast cancer survivors: lessons from the iMove More for Life study. Support Care Cancer. 2017;25(11):3569–85.
4. Bandura A. Health Promotion by social cognitive means. Heal Educ Behav. 2004;31(2):143– 46
5. Craike MJ, Gaskin CJ, Mohebbi M, Courneya KS, Livingston PM. Mechanisms of physical activity behavior change for prostate cancer survivors: a cluster randomized controlled trial. Ann Behav Med. 2018;52(9):798–808.
6. Craike M, Gaskin CJ, Courneya KS, Fraser SF, Salmon J, Owen PJ, et al. Predictors of adherence to a 12-week exercise program among men treated for prostate cancer: ENGAGE study. Cancer Med. 2016;5(5):787–94.
7. Courneya KS, Segal RJ, Reid RD, Jones LW, Malone SC, Venner PM, et al. Three independent factors predicted adherence in a randomized controlled trial of resistance exercise training among prostate cancer survivors. J Clin Epidemiol. 2004;57(6):571–9.
8. Rebar AL, Elavsky S, Maher JP, Doerksen SE, Conroy DE. Habits predict physical activity on days when intentions are weak. J Sport Exerc Psychol. 2014;36(2):157–65.
9. Gardner B, Lally P, Wardle J. Making health habitual: the psychology of “habit-formation” and general practice. Brit J Gen Pract. 2012;62:664–6.
10. Petty RE, Barden J, Wheeler SC. The Elaboration Likelihood Model of persuasion: Developing health promotions for sustained behavioral change. In: DiClemente RJ, Crosby RA, Kegler MC, editors. Emerging theories in health promotion practice and research. San Francisco: Jossey-Bass; 2009. p. 185–2.
11. Nikoloudakis IA, Crutzen R, Rebar AL, Vandelanotte C, Quester P, Dry M, et al. Can you elaborate on that? Addressing participants’ need for cognition in computer-tailored health behavior interventions. Health Psychol Rev. 2018;12(4):437–52.
