# Supplementary material for: Evaluating a web- and telephone-based personalised exercise intervention for individuals living with metastatic prostate cancer (ExerciseGuide): protocol for a pilot randomised controlled trial
Source: Pilot Feasibility Stud. 2021 Jan 11;7:21. doi: 10.1186/s40814-020-00763-2 (PMC7798256; doi:10.1186/s40814-020-00763-2)
Supplement: Supplementary file 2 — Resistance-based exercises prescribed within the ExerciseGuide program. Table detailing all possible resistance training exercises prescribed within the ExerciseGuide program. [file 40814_2020_763_MOESM2_ESM.docx]

Supplementary Table 2: Resistance-based exercises prescribed within the ExerciseGuide program.

| **Upper body exercises** | **Trunk Exercises** | **Lower body exercises** |
| --- | --- | --- |
| Seated Chest Press | Seated Alternate hip flexion | Seated Knee Extension |
| Seated Bicep Curl | Supine Leg Fallout | Seated Hamstring Curl |
| Seated Row | Supine Single-Leg Lift | Sit to Stand |
| Seated Shoulder Press | Supine Single-Leg Lift with Extension | Partial Squat |
| Seated Shoulder Raise | Supine Double Leg Lift | Squat |
| Seated Triceps Extension | Supine Hip Lift | Standing Calf Raise |
| Standing Chest Press | All Fours with Single Leg Extension |  |
| Standing Bicep Curl | All Fours with Single Arm Extension |  |
| Standing Row | All Fours Progression |  |
| Standing Shoulder Press |  |  |
| Standing Shoulder Raise |  |  |
| Incline Push Up |  |  |
