# Supplementary material for: Evaluating a web- and telephone-based personalised exercise intervention for individuals living with metastatic prostate cancer (ExerciseGuide): protocol for a pilot randomised controlled trial
Source: Pilot Feasibility Stud. 2021 Jan 11;7:21. doi: 10.1186/s40814-020-00763-2 (PMC7798256; doi:10.1186/s40814-020-00763-2)
Supplement: Supplementary file 3 — Modified prescription for the last five weeks for individuals unable to comply to at least 80% of resistance exercise prescription. Tabular explanation of modified resistance training exercise prescription for weeks 4-8 for individuals who were unable to comply to at least 80% of resistance exercise prescription. [file 40814_2020_763_MOESM3_ESM.docx]

Supplementary Table 3: Modified prescription for the last five weeks for individuals unable to comply to at least 80% of resistance exercise prescription.

| **Session frequency over first 3 weeks** | **Prescription**  **compliance over first 3 weeks** | **Exercise modifiable variables** | **Week** | | | | |
| --- | --- | --- | --- | --- | --- | --- | --- |
|  |  |  | **4** | **5** | **6** | **7** | **8** |
| > 8  > 8  > 8 | 100%  80-99%  50-79% | Sessions per week | 3 | 3 | 3 | 3 | 3 |
|  |  | Upper/lower body exercises | 3 x 12 | 3 x 10 | 3 x 10 | 3 x 10 | 3 x 8 |
|  |  | Trunk exercises | 3 x 10 | 3 x 10 | 3 x 12 | 3 x 12 | 3 x 12 |
| > 8  6-8 | 0-49%  0-49% | Sessions per week | 2 | 2 | 2 | 2 | 2 |
|  |  | Upper/lower body exercises | 3 x 12 | 3 x 12 | 3 x 10 | 3 x 10 | 3 x 8 |
|  |  | Trunk exercises | 2 x 10 | 3 x 10 | 3 x 10 | 3 x 10 | 3 x 12 |
| 6-8  6-8  6-8 | 100%  80-99%  50-79% | Sessions per week | 2 | 2 | 3 | 3 | 3 |
|  |  | Upper/lower body exercises | 3 x 12 | 3 x 12 | 3 x 10 | 3 x 10 | 3 x 8 |
|  |  | Trunk exercises | 2 x 10 | 3 x 10 | 3 x 10 | 3 x 10 | 3 x 12 |
| 5  5 | 100%  80-99% | Sessions per week | 2 | 3 | 3 | 3 | 3 |
|  |  | Upper/lower body exercises | 3 x 12 | 3 x 12 | 3 x 10 | 3 x 10 | 3 x 8 |
|  |  | Trunk exercises | 3 x 10 | 3 x 10 | 3 x 10 | 3 x 12 | 3 x 12 |
| 5  5 | 50-79%  0-49% | Sessions per week | 2 | 2 | 2 | 2 | 2 |
|  |  | Upper/lower body exercises | 3 x 12 | 3 x 12 | 3 x 10 | 3 x 10 | 3 x 8 |
|  |  | Trunk exercises | 3 x 10 | 3 x 10 | 3 x 10 | 3 x 12 | 3 x 12 |
| 3-4  3-4  3-4 | 100%  80-99%  50-79% | Sessions per week | 2 | 2 | 3 | 3 | 3 |
|  |  | Upper/lower body exercises | 2 x 12 | 3 x 10 | 3 x 10 | 3 x 10 | 3 x 10 |
|  |  | Trunk exercises | 2 x 10 | 3 x 10 | 3 x 10 | 3 x 10 | 3 x 12 |
| 3-4 | 0-49% | Sessions per week | 2 | 2 | 2 | 2 | 2 |
|  |  | Upper/lower body exercises | 2 x 12 | 3 x 12 | 3 x 12 | 3 x 10 | 3 x 10 |
|  |  | Trunk exercises | 2 x 10 | 3 x 10 | 3 x 10 | 3 x 10 | 3 x 12 |
| 0-2  0-2  0-2  0 | 100%  80-99%  50-79%  0-49% | Sessions per week | 2 | 2 | 2 | 2 | 2 |
|  |  | Upper/lower body exercises | 2 x 12 | 2 x 12 | 3 x 12 | 3 x 12 | 3 x 10 |
|  |  | Trunk exercises | 2 x 8 | 2 x 10 | 3 x 10 | 3 x 10 | 3 x 10 |
